# 104 Integrating Addiction Medicine into Burn Care: Optimizing Pain Management in Stimulant-Positive Patients

**DOI:** 10.1093/jbcr/iraf019.104

**Published:** 2025-04-01

**Authors:** Eloise Stanton, Artur Manasyan, Sunnie Wong, Maxwell Johnson, Justin Gillenwater

**Affiliations:** University of Southern California; University of California Keck School of Medicine; Los Angeles General Medical Center; University of California Keck School of Medicine; Los Angeles General Medical Center

## Abstract

**Introduction:**

Stimulant use, such as amphetamines and cocaine, complicates burn care by increasing pain sensitivity, inflammation, and medical complexity, often leading to worse outcomes like prolonged hospital stays and higher complication rates. While addiction medicine offers strategies to address substance use disorders, its role in burn care is not well-defined. This study aims to assess the impact of stimulant use on burn patient outcomes and characterize the involvement of addiction medicine in managing these patients.

**Methods:**

This retrospective cohort study analyzed burn patients treated at a Level 1 trauma center from 2015 to 2024. Stimulant-positive patients were identified through urine toxicology screens, and primary outcomes included hospital length of stay, burn severity, and complications. The use of addiction medicine consultations was also examined. Statistical analyses were conducted using Stata 17.0, with significance set at p< 0.05.

**Results:**

Out of 3,403 burn patients (34% female, 66% male, with a mean age of 39.2 ± 22.8 years), 572 patients (16.8%) had positive urine toxicology screens for stimulants. Stimulant-positive patients had significantly longer hospital stays compared to stimulant-negative patients (17.7 vs. 10.7 days, p< 0.001), more severe burns (p=0.001), and a higher incidence of complications (15.6% vs. 11.5%, p=0.006). These patients were also more likely to suffer inhalation injuries (28.5% vs. 21.1%, p=0.007) and required more surgical interventions (p=0.026). Additionally, stimulant-positive patients had longer ICU stays and more days on mechanical ventilation. Despite these risks, only 12.6% (72 patients) of stimulant-positive burn patients received an addiction medicine consultation during their hospitalization. However, the use of addiction medicine consultations increased over time, rising from three consults in 2015 to 42 in 2023.

**Conclusions:**

Stimulant-positive burn patients face more severe injuries and worse outcomes, yet addiction medicine remains underutilized in their care. These findings highlight the critical need to integrate addiction medicine into burn care protocols to better manage pain and address substance use. Early involvement of addiction medicine teams should be prioritized to improve outcomes for this high-risk group. Further research should explore barriers to consultation and the impact of addiction medicine on long-term recovery.

**Applicability of Research to Practice:**

This study emphasizes the need to incorporate addiction medicine into standard burn care protocols for patients with stimulant use disorders to improve pain management and reduce complications. Early consultation with addiction specialists can lead to more comprehensive treatment, enhancing both recovery outcomes and long-term rehabilitation for this high-risk population.

**Funding for the Study:**

N/A

